# Quantifying immediate and delayed effects of anthelmintic exposure on ecosystem functioning supported by a common dung beetle species

**DOI:** 10.1371/journal.pone.0182730

**Published:** 2017-08-11

**Authors:** Paul Manning, Sarah A. Beynon, Owen T. Lewis

**Affiliations:** 1 Department of Plant, Food, and Environmental Sciences, Faculty of Agriculture, Dalhousie University, Truro, Nova Scotia, Canada; 2 University of Oxford, Department of Zoology, Oxford, United Kingdom; 3 Dr Beynon’s Bug Farm, Lower Harglodd Farm, St. David’s, Pembrokeshire, United Kingdom; University of Pretoria, SOUTH AFRICA

## Abstract

Dung beetles (Coleoptera: Scarabaeoidea) support numerous ecosystem functions in livestock-grazed pastures. Exposure to veterinary anthelmintic residues in livestock dung can have lethal and sublethal effects on dung beetles, and can reduce rates of dung removal, but the immediate and longer-term consequences for other dung beetle mediated functions have rarely been studied. We investigated the consequences of anthelmintic exposure on survival of the dung beetle *Aphodius fossor* and its delivery of four ecosystems functions that underpin pasture production: dung removal, soil fauna feeding activity, primary productivity, and reduction of soil compaction. We tested whether anthelmintic exposure had immediate or delayed effects on these functions individually and simultaneously (i.e., ecosystem multifunctionality). We found no evidence that ivermectin residues had a lethal effect on adult beetles. For dung removal, we found a significant interaction between the timing of exposure and functioning: while dung removal was impaired by concurrent exposure to high levels of ivermectin, functioning was unaffected when beetles that had been exposed previously to the same concentration of anthelmintic later interacted with untreated dung. Other ecosystem functions were not affected significantly by anthelmintic exposure, and there was no evidence to suggest any persistent impact of anthelmintic exposure on ecosystem multifunctionality. While anthelmintic residues remain a significant threat to dung beetle populations, for adult beetles, we found no evidence that residues have detrimental consequences for ecosystem functioning beyond the immediate point of exposure.

## Introduction

Globally, more than 80% of agricultural land is used for grazing livestock [1]. This production method relies on a suite of ecosystem functions. The most obvious of these is primary production, which in turn is underpinned by supporting functions such as nutrient cycling [2], improved physical properties of soil [3], and the control of herbivorous pests [4].

Many of these ecosystem functions are supported by soil invertebrates including dung beetles (Coleoptera: Scarabaeoidea) [5,6]. Most dung beetles feed on livestock dung as larvae and adults [7] and some species incorporate dung into the soil when provisioning their offspring [8]. Collectively, these actions remove dung from the pasture surface, which can limit the spread of gastrointestinal parasites [9], improve soil permeability [10,11], increase primary productivity [11,12], and stimulate more rapid decomposition of plant litter [13,14].

Dung beetles, and the ecosystem functions that they mediate, are vulnerable to perturbations associated with agricultural management [15]. For example, soft-bodied larvae in the soil are unlikely to survive cultivation practiced in short-term grazing leys [16] and the removal of hedgerows can cause local extinctions of species with narrow thermal niches [15].

Dung beetles are also vulnerable to veterinary anthelmintic residues in livestock dung [17,18]. Veterinary anthelmintics are routinely administered to livestock to manage internal parasites. The most widely-used class of anthelmintics is the macrocyclic lactones [19]. These compounds bind to glutamate-gated chloride channels, causing hyperpolarisation of nerve cells which leads to rapid paralysis and death in parasites and many other invertebrates [20]. Macrocyclic lactones are poorly metabolized and are excreted in dung and urine [19]. Dung beetles are exposed to residues when feeding on dung from treated animals: these residues cause a suite of lethal [21,22] and sublethal effects including reduced fecundity [23,24], weaker muscles [25], and prolonged development [23].

Through a combination of these lethal and sublethal effects, anthelmintic residues are thought to negatively affect ecosystem functioning supported by dung beetles. Exposure to these residues has been shown repeatedly to reduce dung removal rates [21,26]. However, dung removal is not always correlated with other functions supported by dung beetles [13], raising the possibility that the effect of anthelmintics may be stronger, or weaker, for other ecosystem functions. The effects may differ further when considering multiple ecosystem functions simultaneously—a phenomenon known as ecosystem multifunctionality [27].

A second knowledge gap associated with anthelmintic residues is the delayed impact of exposure on ecosystem functioning occurring after exposure (herein, ‘successive functioning’). Published literature on the effect of anthelmintic exposure on functioning considers impacts only at the source of exposure (i.e. the dung pat containing anthelmintic residues). However, beetles will typically disperse among multiple dung pats as adults [28], potentially experiencing varying levels of exposure. The negative impacts of anthelmintic residues may therefore persist even when dung beetles later interact with anthelmintic-free dung, but this has not previously been investigated.

Here, we investigate how exposure to residues of ivermectin (a widely used macrocyclic lactone anthelmintic) affects four ecosystem functions mediated by the dung beetle *Aphodius fossor* L., a species known to play a key role in promoting multiple ecosystem functions [29]. In addition to quantifying the concurrent effect of residue exposure on functioning, our experiment allows us to investigate the delayed lethal and sublethal effects of anthelmintic exposure on ecosystem functioning. We ask: **i)** Does exposure to anthelmintic residues have lethal effects on *A*. *fossor*? **ii)** Does exposure to anthelmintic residues affect the ability of *A*. *fossor* to support individual functions concurrently with exposure? **iii)** Does measuring functioning solely at the source of exposure underestimate or overestimate functional impairment associated with anthelmintic use? and **iv)** Do anthelmintic residues cause persistent declines in ecosystem multifunctionality beyond the source of initial exposure?

## Methods

### Overview

**Fig 1:**
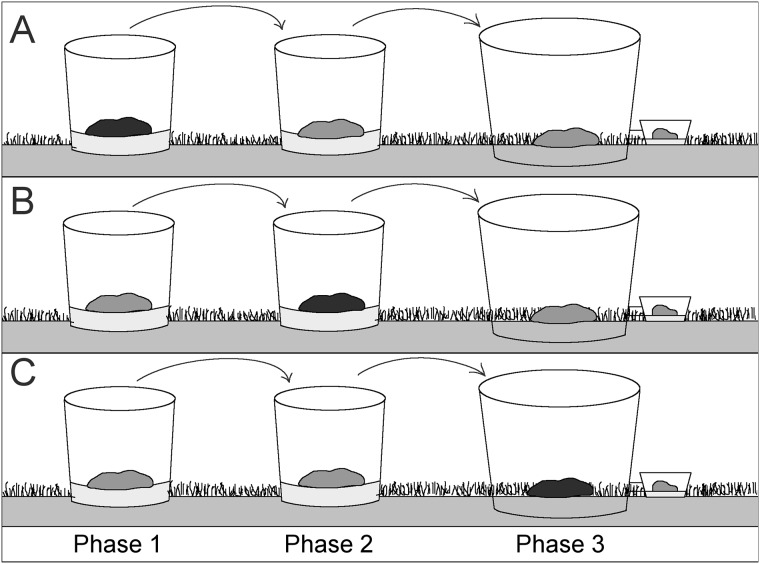
Schematic of the experimental design. All dung beetles used within the experiment were moved through a series of three enclosures, spending one week in each Phase. Black dung indicates the period of the experiment when the anthelmintic was added (or not added in the controls) to dung. All ecosystem functions were measured during the final part of the experiment (Phase 3). To manipulate the time between exposure and measurement of functioning, beetles were exposed to ivermectin (except in the controls) during either Phase 1, Phase 2, or Phase 3, which corresponded to ‘exposure-delays’ of two-weeks prior to (A), one-week prior to (B), or concurrently alongside (C) the enclosure where functions were measured (Phase 3). The experiment was replicated with high anthelmintic residues (500 ppb ivermectin), low anthelmintic residues (125 ppb ivermectin), or controls without anthelmintic. Enclosures used for Phase 1 and Phase 2 were fully sealed, while enclosures used for Phase 3 were open at the bottom, and dug into the soil to allow free colonisation by soil invertebrates.

We conducted our experiment between June 7^th^ and September 5^th^ 2016 in a grassy field at the John Krebs Field Station (Wytham, Oxfordshire, OX2 8QJ, UK). Our design was based on systematically moving groups of dung beetles through a series of three enclosures over a three-week period. Adult dung beetles fed in each enclosure for one week, with each enclosure being deemed a ‘Phase’. During one of the three Phases, dung beetles were exposed to dung containing either high anthelmintic residues (500 ppb ivermectin), low anthelmintic residues (125 ppb ivermectin), or controls without anthelmintic. The high concentration corresponds to peak faecal concentration of ivermectin of cattle treated with injectable formulations, and the low concentration corresponds to concentrations observed 10 days after treatment [30], when anthelmintic exposure is thought to pose relatively limited toxicological risk to dung beetles [31]. Staggering the Phase when beetles were exposed (Fig 1) allowed us to compare the effect of anthelmintic residues on beetles exposed either two weeks before- (Fig 1A), one week before (Fig 1B), or alongside our measures of functioning (Fig 1C). This delay between exposure and measures of functioning is herein referred to as ‘exposure-delay’. Each of the treatment combinations (3 exposure-delays x 3 residue concentrations) was replicated 8 times, giving 72 replicates in total.

### Enclosure design

Enclosures used for the Phases 1 and 2 of the experiment consisted of 5 L plastic buckets, filled with 5 cm of builders’ sand, with six 3 mm holes cut in the bottom for drainage. Enclosures used for Phase 3 were 12 L black plastic buckets with the bottoms removed and dug into the ground to a depth of 8-cm [13]. This allowed us to measure functions delivered by dung beetles and other soil fauna, which could colonise from the underlying soil. Enclosures were arranged in 7 x 8 m grid, maintaining a 1 m spacing between the centre-point of each enclosure and its neighbour. This small spatial scale was chosen to minimise spatial variation in the physical and chemical properties of soil across replicates. Replicates were allocated to random positions on this grid.

We collected dung for the enclosures from organically managed beef cattle, raised onsite at the Farm Animal Initiative (Wytham Farm, Oxfordshire, OX2 8QJ, UK). Cattle had not been treated with anthelmintics for at least 4 months prior to dung collection. At the time of dung collection, cattle were grazing on semi-improved pasture dominated by perennial ryegrass (*Lolium perenne* L.) and white clover (*Trifolium repens* L.). All dung was collected fresh (within 3 hours of defecation), frozen at -20°C for at least 48 hours to kill any invertebrates within it, and thawed before use. Dung was homogenised for several minutes so that dung pat consistency was standardised amongst enclosures.

To expose dung beetles to anthelmintic residues, we spiked dung with ivermectin. Following Römbke *et al*. (2009), we prepared dilute solutions of ivermectin (5mg/mL Molemec pour-on, Mole Valley Farmers, South Molton, Devon EX36 3LH, UK) in acetone at two concentrations: 275 ppm solution (high) and 68.75 ppm solution (low). A solution of pure acetone was used as a control. A third party coded the solutions by allocating a unique colour to each randomly, which blinded researchers to treatment levels for the duration of the experiment.

Each pat was formed after homogenising a 1-mL aliquot of the anthelmintic solution into 550 g of dung for 60 seconds. Addition of the high and low concentration solutions to dung resulted in final ivermectin concentrations of *c*. 500 and 125 ppb (wet dung mass), respectively. Control enclosures not subject to treatment with anthelmintic residues were treated with 1 mL of pure acetone.

We formed dung pats within the enclosures using a 12-cm diameter circular frame. Dung pats were placed on a 2-cm wire grid. Each enclosure was then covered by 2-mm white fine mesh to prevent additional insect colonisation and a 1-mm blue mesh to provide shade. For each Phase, we postponed the addition of beetles to enclosures for three days following dung pat formation, allowing time for acetone to volatise [32], and for dung to reach a more favourable condition for *A*. *fossor*, which prefers older dung [33].

Dung beetles used in the experiment were collected from two farms near to the experimental site (Wytham Farm: 51°46'N, 001°18'W, Medley Manor Farm: 51°45' N, 001°16'W.), where organically-managed beef cattle were grazing semi-improved pasture. Beetles were hand collected from dung, stored in mixed-sexed terraria, and fed cattle dung free from anthelmintic residues before being used in the experiment.

We determined the sex of each beetle and added two males and two females to each enclosure. The experiment began on June 7^th^, 2016 (Day 1) by adding beetles to the first enclosures (Fig 1, Phase 1). We allowed beetles to feed for 7 days before removing beetles through a combination of hand-sorting and flotation (submerging dung pats and soil in water, and capturing beetles which floated to the surface). The numbers of live and dead beetles were recorded and all living beetles were immediately transferred to the corresponding enclosure (Fig 1, Phase 2). Seven days later, on Day 14, the beetles were removed as before and added to the final enclosure (Fig 1, Phase 3). All measures of functioning were taken six weeks later (Day 56), corresponding to the egg-to-pupa development period reported for *A*. *fossor* [34].

Removing beetles through flotation at the end of Phase 3 would have prevented our measurement of ecosystem functioning. Thus, to estimate beetle survival from Phase 3, we attached an emergence trap baited with cattle dung (free from anthelmintic residues) to each enclosure on Day 21. Emergence traps consisted of translucent 750-mL plastic containers attached to each of the enclosures with a short length of clear 2-cm plastic tubing (Fig 1). Each trap was filled with approximately 1-cm of builders’ sand and baited with 150-g of previously frozen and thawed cattle dung from the same source used in the experiment. An additional 50-g of dung was added one week later, and original baits were refreshed concurrently by disturbing the dung’s crust with a knife. As beetles move between multiple dung pats as adults [28], provision of fresh dung encouraged movement from the enclosure into the emergence trap. Presence of *A*. *fossor* individuals in dung has been shown to influence aggregation [35], so beetles were removed from the traps every two to three days for two weeks until three consecutive evaluations of emergence yielded no additional beetles.

### Response variables

We measured five response variables during the experiment: beetle survival, feeding activity of soil invertebrates, dung removal, soil compaction, and primary productivity. *Beetle survival* was calculated as the total number of adult beetles collected from emergence traps between Days 21–34.

We measured the *feeding activity of soil invertebrates* (for brevity, referred to hereafter as *feeding activity)* using the bait lamina test (Terra Protecta GMbH, Berlin, Germany). Bait lamina are PVC strips (1 mm x 6 mm x 120 mm) with 1.5 mm diameter holes, spaced at 5 mm intervals. Holes are filled with a standardized bait of powdered cellulose, wheat bran and charcoal (70:27:3). Bait lamina are inserted into the soil, where soil invertebrates can access them freely and consume the baits; which allows an estimate of ‘feeding activity’ [36]. Six weeks after introducing beetles to Phase 3 (Day 56) we inserted five bait lamina strips through each dung pat [13], positioned so that the uppermost hole was located immediately beneath the soil surface. Thirty-six strips were placed alongside the experiment and a subset was checked every two days until between 30–50% feeding activity was observed. In our experiment, this occurred after 12 days. Bait lamina were removed from the ground on Day 68 (August 13^th^, 2016) and feeding activity was assessed by scoring each bait as ‘consumed’ or ‘intact’ by viewing the strips against a light source.

Immediately after removing bait lamina on Day 68, we began measuring all remaining functions. We measured *dung removal* by lifting dung pats from the enclosures using the wire grid. All dung was immediately placed in an aluminium weigh boat and dried for 48 hours at 75°C [37]. Dung removal was calculated as the dry mass (g) of dung remaining, subtracted from the mean dry mass (91 g) of 550-g dung pats with all invertebrates excluded (n = 3).

Under the initial location of the dung pat we hammered three lengths of 5.5-cm diameter PVC pipe into the soil to a depth of 5-cm. We removed the intact samples, each contained within an individual pipe using a trowel. We assessed *soil compaction* using a measure of bulk density, by placing the first sample into an aluminium weigh boat and drying the sample for 48 hours at 80°C. Soil bulk density (g·cm^-3^) was calculated as the dry mass of soil solids, divided by the core volume.

We measured *primary productivity* by planting perennial ryegrass seeds into the two remaining soil cores sampled from the enclosures. Soil samples were homogenised, and a 185-mL subsample was added to a 250-mL plant pot. We scattered 1-mL of tetraploid perennial ryegrass (Cotswold Seeds, Moreton-in-Marsh, Gloucestershire, GL56 0JQ, UK) on the soil surface, and covered the seed with 15-mL of soil from the same sample. We placed the pots in an unheated greenhouse and watered all pots regularly and equally. Three weeks after planting, we harvested above-ground biomass by clipping plants at the soil surface, drying at 75°C for 48 hours (Zhao *et al*., 2013), and weighing to the nearest 0.1 g.

### Analysis

#### i) Does exposure to anthelmintic residues have lethal effects on *A*. *fossor*?

For each ‘exposure-delay’, we tested how exposure to anthelmintic residues influenced dung beetle mortality by modelling emergence (proportion emerged) as a function of ivermectin concentration (high, low, control) using analysis of variance (ANOVA). Model residuals met assumptions of homogeneity and normality and thus data were left untransformed.

#### ii) Does exposure to anthelmintic residues affect the ability of *A*. *fossor* to support individual functions concurrently with exposure?

We tested whether anthelmintic residues at the site of exposure affected the delivery of individual functions using the subset of our data where exposure occurred concurrently alongside measures of functioning (exposure-delay = 0 weeks, Fig 1C). We modelled each individual measure as a function of anthelmintic dose (high, low, control) using ANOVA. Model residuals met assumptions of homogeneity and normality, and thus data were left untransformed. When a significant effect of treatment was found, treatment level means were compared using Tukey’s Honestly Significant Differences (Tukey’s HSD), and effect sizes were estimated using Cohen’s D.

#### iii) Does measuring functioning solely at the source of exposure underestimate or overestimate functional impairment associated with anthelmintic use?

We tested whether solely considering the immediate effect of anthelmintic residues underestimates or overestimates functional losses associated with exposure, using analysis of covariance. We modelled individual functional measures as a function of anthelmintic concentration (high, low, control), exposure-delay (0 weeks, 1 week, 2 weeks), and the interaction between anthelmintic concentration and exposure-delay.

#### iv) Does anthelmintic exposure cause persistent declines in ecosystem multifunctionality beyond the source of initial exposure?

We tested whether the effect of ivermectin exposure on multifunctionality acted mainly at the source of exposure, or whether continued lethal and sublethal effects resulted in loss of multifunctionality using the ‘exceeding thresholds approach’ [38]. This method, developed originally to describe the relationship between diversity and multifunctionality, works by estimating a linear relationship between a continuous independent variable (exposure-delay ranging from 0–2 weeks), and the number of ecosystem functions occurring at or above a given threshold (0–4 functions). Estimates were achieved using a linear model with a quasi-binomial error structure.

We described the relationship between exposure-delay and multifunctionality for each of the three exposure levels (high, low, control), to the full threshold limits under which our models would converge (10–87%). To visualise the relationship between exposure-delay and multifunctionality, all slopes and their 95% confidence intervals were plotted across this full range.

If exposure to anthelmintic residues cause a delayed impact on functioning, we would expect to see a negative relationship between exposure-delay and the number of functions surpassing thresholds in both the low and high residue treatments. We would not expect to see any significant relationship between exposure-delay and multifunctionality under control conditions. When differences are found (i.e. the confidence intervals do not overlap with y = 0), a number of metrics can be derived from the curve to facilitate comparisons of multifunctionality amongst treatments [38].

The statistical package R 3.1.1 [39] was used for all analyses. The exceeding thresholds approach to assessing multifunctionality was analysed using the multifunc package [38] All figures were created using the ggplot2 package [40]. Methodology and code were pre-registered on the Open Science Framework before beginning the experiment [41], and are available online.

## Results

### i) Does exposure to anthelmintic residues have lethal effects on *A*. *fossor*?

**Fig 2:**
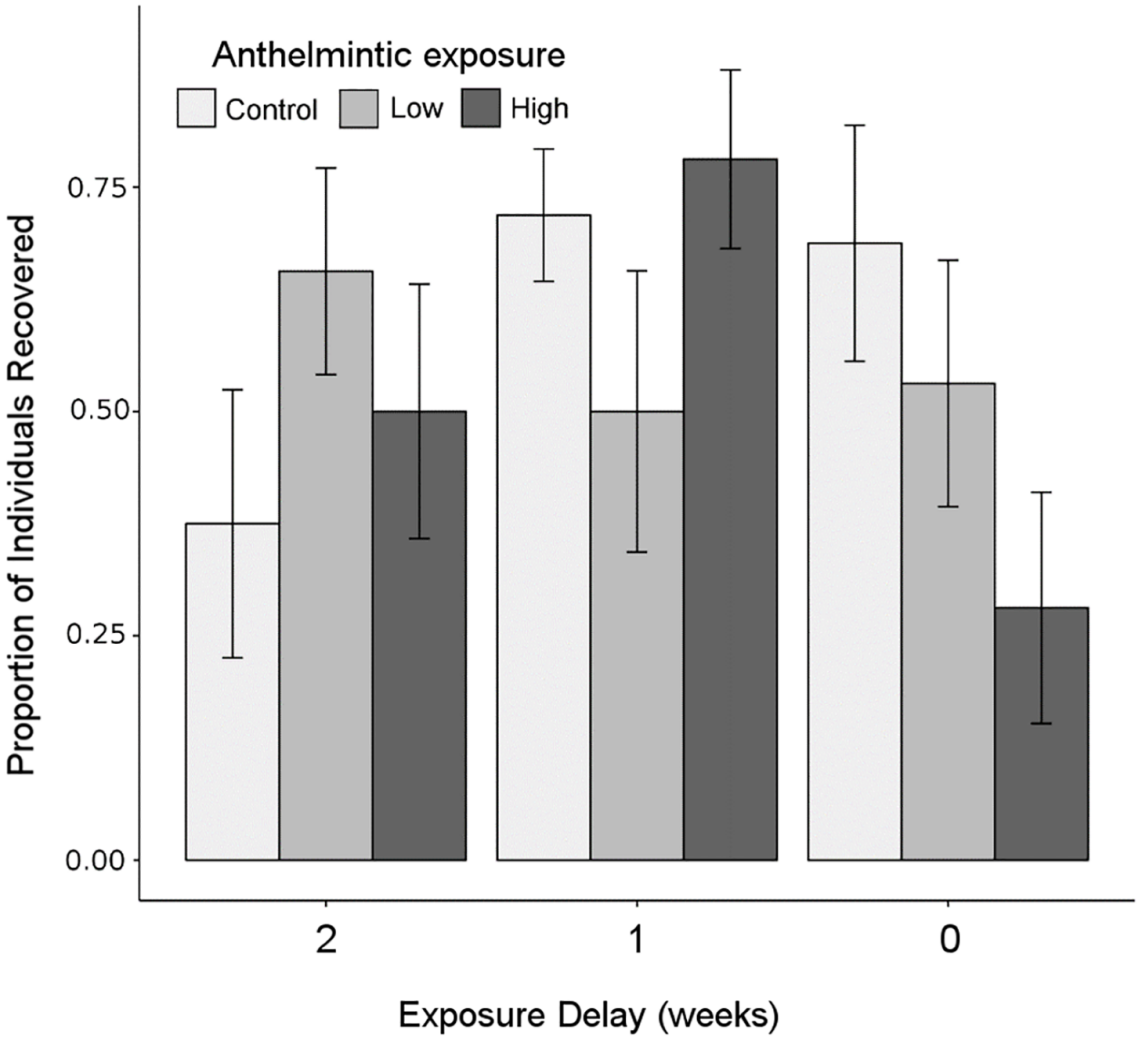
Dung beetle survival as a function of anthelmintic exposure. Dung beetle survival was not significantly affected by anthelmintic exposure. Means (bars) are calculated using the number of adult *Aphodius fossor* dung beetles entering emergence traps at the end of the experiment (Phase 3). Means are grouped based on the exposure-delay. Bars represent treatment means with standard errors.

Beetle survival was not significantly affected by anthelmintic residues two weeks after (F_2,21_ = 1.07, *P* = 0.36), one week after (F_2,21_ = 1.64, *P* = 0.21), or concurrently alongside (F_2,21_ = 2.39, *P* = 0.11) exposure (Fig 2).

### ii) Does exposure to anthelmintic residues affect the ability of *A*. *fossor* to support individual functions concurrently with exposure?

**Fig 3:**
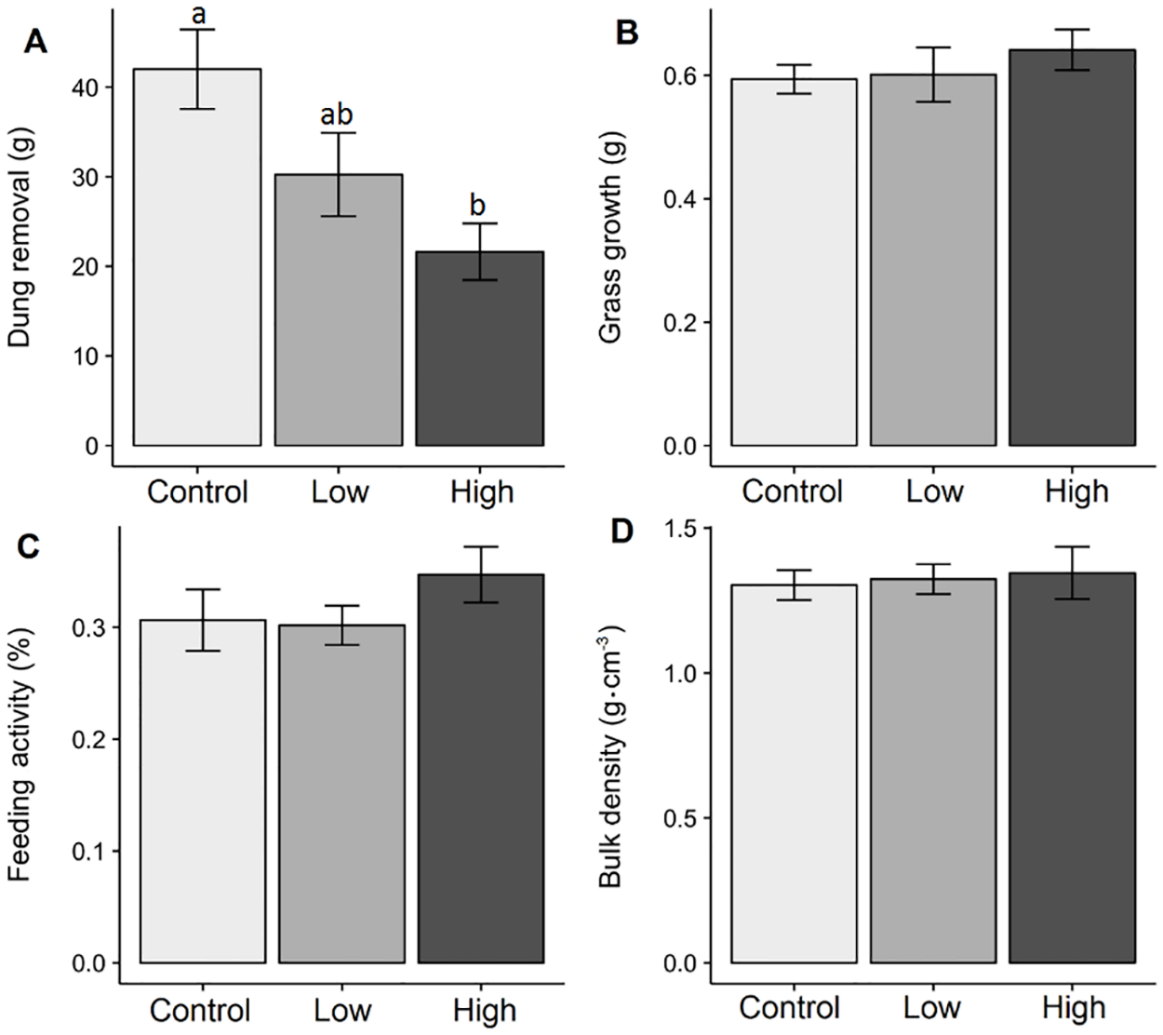
The effect of concurrent ivermectin exposure on four ecosystem functions supported by the dung beetle *Aphodius fossor*. Each panel shows a different ecosystem function: dung removal (A), primary productivity (B), feeding activity (C), and soil compaction (D). Bars represent treatment means with standard errors. In panel A, sharing of lowercase letter labels indicate that differences between treatment means are not significant, at a level of: α = 0.05.

For concurrent exposure, dung removal was significantly affected by anthelmintic residue level (F_2,21_ = 6.13, *P* < 0.01), with approximately twice the level of dung removed from controls relative to dung with high levels of anthelmintic residues (Control: 42.0 ± 4.1-g, High exposure: 21.6 ± 2.95-g mean ± SE, Cohen’s D = -2.00, Fig 3A). No significant effect of concurrent anthelmintic exposure on functioning was observed for primary productivity (F_2,21_ = 0.55, *P* = 0.59), feeding activity (F_2,21_ = 1.11_,_ *P* = 0.35)_,_ or soil bulk density (F_2,21_ = 0.10, *P* = 0.91).

### iii) Does measuring functioning solely at the source of exposure underestimate or overestimate functional impairment associated with anthelmintic use?

**Fig 4:**
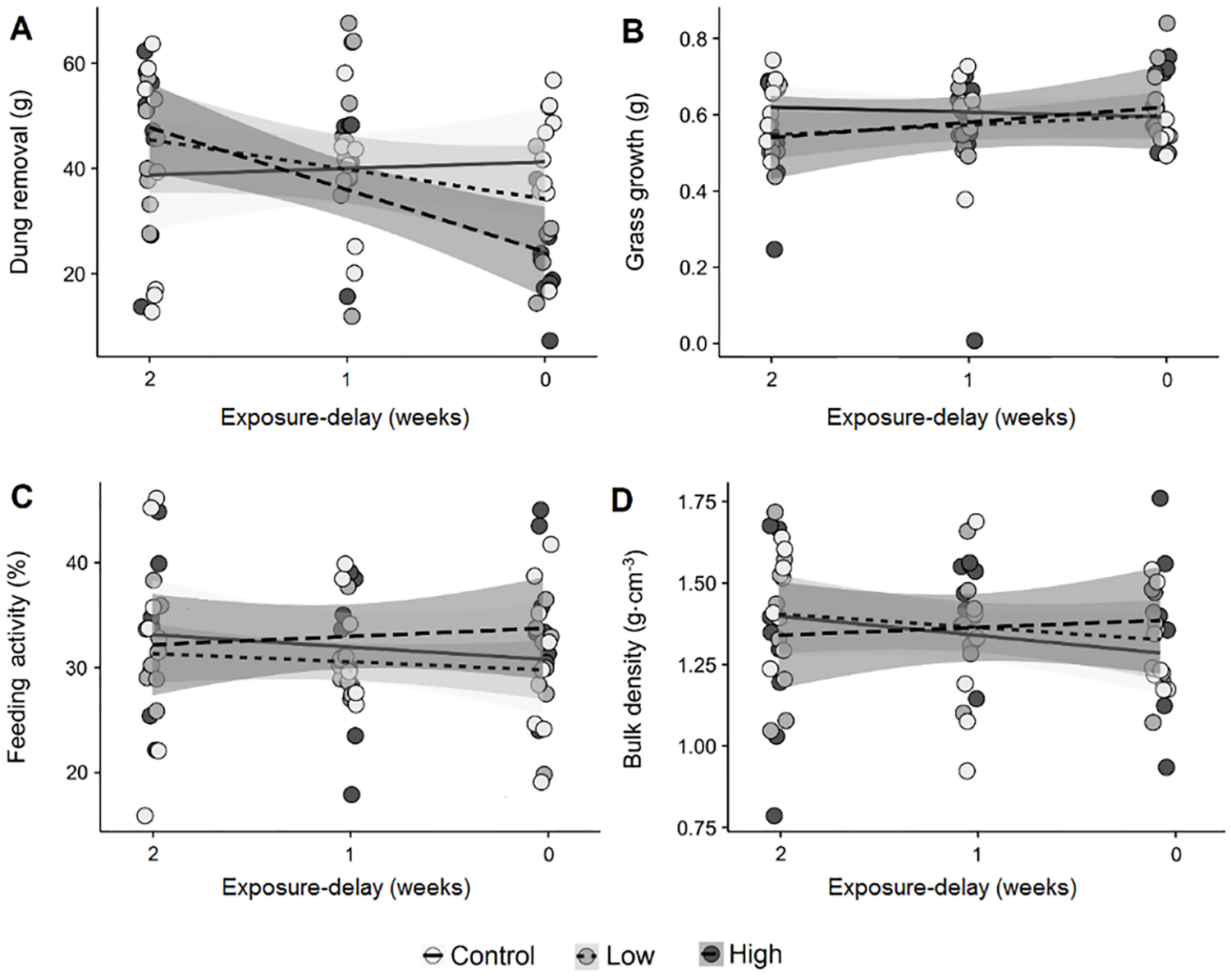
The interacting effects of anthelmintic residues and exposure-delay on four ecosystem functions supported by dung beetles. Measures of dung removal (A), primary productivity (B), feeding activity (C) and soil compaction (D), are shown as a function of species richness and perturbation with the anthelmintic ivermectin. Relationships between each function and the timing of exposure are plotted along with 95% confidence intervals at each level of anthelmintic exposure. Light-grey-filled circles with unbroken lines represent anthelmintic-free controls, grey-filled circles with dotted trend lines represent low exposure (125 ppm), and dark-grey-filled circles with dashed trend lines represent high exposure (500 ppm).

For dung removal, we found a significant interaction between exposure-delay and level of anthelmintic residues (Table 1). Differences in dung removal for past exposure (Phases 1 and 2) were negligible amongst residue levels. However, for concurrent exposure (Phase 3), dung removal was significantly lower at high anthelmintic exposure in comparison to controls (Fig 4A). Neither exposure-delay, exposure levels, or their interaction significantly explained any variation in the other three ecosystem functions considered.

**Table 1:** Summary of linear models describing the relationship between exposure-delay (Delay), and the concentration of anthelmintic residues (Residues) in cattle dung.

	F	*P*-value
**Dung removal**		
Residues	F_2,66_ = 0.60	0.55
Delay	F_1,66_ = 6.65	0.01
Residues*Delay	F_2,66_ = 3.25	0.05
**Primary productivity**		
Residues	F_2,66_ = 0.55	0.57
Delay	F_1,66_ = 1.02	0.31
Residues*Delay	F_2,66_ = 0.81	0.45
**Bait Lamina**		
Residues	F_2,66_ = 0.81	0.45
Delay	F_1,66_ = 0.17	0.68
Residues*Delay	F_2,66_ = 0.40	0.67
**Bulk Density**		
Residues	F_2,66_ = 0.10	0.91
Delay	F_1,66_ = 0.67	0.42
Residues*Delay	F_2,66_ = 0.66	0.52

### iv) Does anthelmintic exposure cause persistent declines in ecosystem multifunctionality beyond the source of initial exposure?

**Fig 5:**
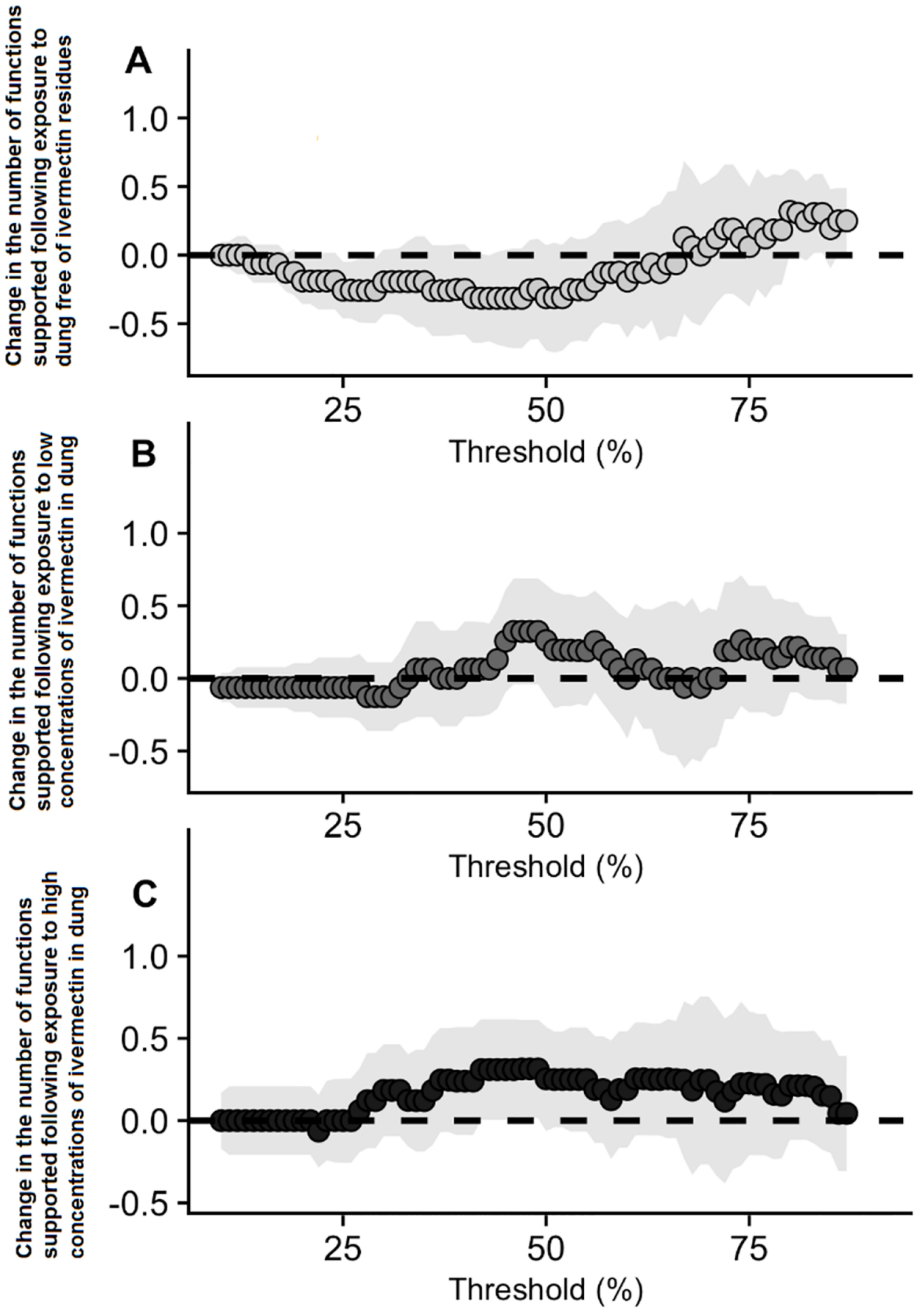
The relationship between the exposure timing of the anthelmintic ivermectin and ecosystem multifunctionality. Relationships between exposure timing and multifunctionality at control (A), low (B) and high (C) levels of anthelmintic exposure. Thresholds span between 10–87% of the observed maxima. Confidence Intervals (95%) surrounding the slope estimates indicate whether the intervals contain zero. In no case did we find any evidence of a strong loss or gain of multifunctionality associated with anthelmintic exposure.

Exposure-delay did not have a marked effect on multifunctionality. In the absence of any anthelmintic residues, exposure-delay briefly had a positive influence on multifunctionality when thresholds reached 80% of their maxima (Fig 5A), although this almost certainly represents a false-positive from random noise. In the case of low exposure levels (Fig 5B) and high exposure levels (Fig 5C), the number of functions pushed above or below the corresponding thresholds was never significantly different from zero.

## Discussion

Overall, we found little immediate impact of ivermectin exposure in directly reducing *A*. *fossor* survival (Fig 2). Dung removal was significantly impaired by concurrent exposure to high levels of ivermectin. However, no other measures of functioning provided by *A*. *fossor* were affected (Fig 3). We found no evidence to suggest that past exposure had any effect on future delivery of individual functions (Fig 4, Table 1) or for ecosystem multifunctionality (Fig 5). This suggests that considering functioning solely at the site of exposure (Fig 3) might overestimate the detrimental effects of anthelmintic residues on adult dung beetle functioning. However, ivermectin residues are known to negatively affect dung beetles up to two weeks following subcutaneous treatment [30], and up to 20 weeks following treatment with a sustained release bolus [42]; thus the concurrent exposure period is in itself significant. Furthermore, we are focussing on a species in which ivermectin effects on mortality are low, relative to other dung beetle species [21]. Lastly, because we moved beetles to their new habitat patch in our experiment, rather than requiring them to disperse, our experiment may have underestimated negative functional consequences if anthelmintic exposure affects dispersal, perhaps by inhibiting locomotor performance [25].

Recent research has demonstrated that low-dose ivermectin exposure causes a number of sublethal effects in the dung beetle *Scarabaeus cicatricosus* Lucas [25]. However, in these experiments adult beetles were continually supplied with spiked dung. Such prolonged exposure is unlikely to reflect typical field conditions, unless anthelmintics are administered through sustained-release boluses [42], or beetles move amongst groups of livestock treated at different times. If anthelmintic residues have little impact on dung beetles beyond the site of exposure, ensuring the availability of anthelmintic-free dung through selective or split-treatment of herds (retaining refugia of ‘clean’ dung) could be useful in limiting the non-target impacts of anthelmintic residues on dung-dwelling insects [43]. In addition, maintaining refugia can slow the onset of anthelmintic resistance in parasite populations [44].

In the case of individual functions, we found that dung removal was significantly impaired by anthelmintic residues at high concentrations (Fig 4A), consistent with previous studies [11,21,45]. We expect this was caused by a combination of lethal and sub-lethal effects on dung beetle larvae, which drive dung removal by bulk-feeding on undigested plant material and microbial biomass [46]. In contrast, adult beetles filter out large particles and consume the liquid fraction of dung [46]. As we estimated dung removal with a measure of dry mass, we likely considered larval feeding only. All other functions considered were not impacted by either high or low levels of anthelmintic residues in dung, which is broadly consistent with previous results [11]. While dung removal is a useful measure in estimating baseline levels of ecosystem functioning [5], it seems to be poorly correlated with other functions of interest, and may not reflect loss of related functions, or ecosystem multifunctionality.

Consistent with Beynon *et al*. (2012), we found no significant effect of anthelmintic exposure on survival of adult *A*. *fossor* beetles (Fig 2). However, in both cases, the ability to detect lethal effects may have been obscured by using field-captured rather than newly-emerged beetles. In order to build fat reserves, newly emerged dung beetles consumer greater quantities of dung, thus increasing their exposure to anthelmintic residues. Using laboratory reared beetles of a constant age, or considering the fate of developing larvae [42,47,48] would reflect more fully the non-target risk anthelmintics pose to dung beetles and other insects.

A limitation of our experiment is that we considered only one dung beetle species, albeit a functionally important one. *Aphodius fossor* is often common at intensively managed sites [15] suggesting that it may have relatively low sensitivity to perturbations such as anthelmintics. Contrasting functional consequences may be seen for dung beetle species that are more sensitive to ivermectin [49]. Furthermore, dung beetles represent only a small portion of the diverse ecological community present in dung pats [7,50]. Coprophagous flies are typically the most abundant taxa in livestock dung [50], and have higher sensitivity to chemical residues than dung beetles [47]. Although these flies have been shown to support dung removal levels comparable to small-bodied temperate dung beetles [51], little attention has been given to the ecosystem functioning they support.

While we cannot be certain that mixing fully homogenized ivermectin throughout the dung, we used widely referenced methods [32] and the limited evidence available suggests in field conditions that anthelmintic concentrations are highly variable in different parts of the dung pat [52]. Future research would benefit from a more comprehensive understanding of the heterogeneity of anthelmintic residues, which can be quantified using liquid chromatography-tandem mass spectrometry [53].

Because functioning was measured in mesocosms where earthworms and other invertebrates were allowed to colonise dung from the underlying soil, loss of dung beetle functioning associated with anthelmintic exposure may have been masked or buffered by soil fauna [5]. Similarly, variation in the physiochemical properties of soil may also have made identifying difference among treatments difficult, despite our experiment being set-up over a relatively small spatial scale. Closed mesocosms, as used by Beynon *et al*. [21], would allow effects on dung removal to be estimated more precisely, but would not allow other functions to be measured. This trade-off between precision and perspective is inevitable; however, we believe that our enclosure design allowed for more conservative estimates of functional losses and gives context to the importance of dung beetles relative to other taxa in underpinning ecosystem functioning in pastures.

Perhaps the biggest challenge in understanding the non-target consequences of livestock parasite control for ecosystem functioning is the difficulty of conducting experiments over large spatial scales and over multiple beetle generations. Our approach, which considered the functional losses associated with past exposure to anthelmintic residues, goes beyond the typical direct effects but does not consider impacts of anthelmintic use on multiple beetle generations. Additionally, under intensive systems, dung beetles may be exposed to many different anthelmintic (and indeed ectoparasiticide) residues for longer periods [42], and at higher concentrations [30] than those we considered here. Achieving a better understanding of patterns of anthelmintic use, the long-term effects of residues on dung beetles, and the chemical sensitivity of common species to anthelmintics[49]would be useful in informing models which predict losses of ecosystem functioning caused by the non-target impacts of livestock parasite control.

## Supporting information

S1 Table Raw data from the experiment. (CSV)
